# Clinical correlates of “pure” essential tremor: the TITAN study

**DOI:** 10.3389/fneur.2023.1233524

**Published:** 2023-08-23

**Authors:** Roberto Erro, Giulia Lazzeri, Angelo Fabio Gigante, Andrea Pilotto, Luca Magistrelli, Matteo Bologna, Carmen Terranova, Enrica Olivola, Carlo Dallocchio, Vincenzo Moschella, Francesca Valentino, Francesca Di Biasio, Alessandra Nicoletti, Rosa De Micco, Livia Brusa, Cristiano Sorrentino, Angela Matinella, Salvatore Bertino, Giulia Paparella, Nicola Modugno, Elena Contaldi, Alessandro Padovani, Alessio Di Fonzo, Marialuisa Restaino, Paolo Barone, TITAN study group, Giulia Franco

**Affiliations:** ^1^Department of Medicine, Surgery and Dentistry “Scuola Medica Salernitana”, Neuroscience Section, University of Salerno, Baronissi, SA, Italy; ^2^Foundation IRCCS Ca' Granda Ospedale Maggiore Policlinico, Neurology Unit, Milan, Italy; ^3^Department of Medical Sciences and Public Health, Section of Neurology, San Paolo Hospital, Bari, Italy; ^4^Neurology Unit, Department of Clinical and Experimental Sciences, University of Brescia, Brescia, Italy; ^5^Department of Translational Medicine, Movement Disorders Centre, Neurology Unit, University of Piemonte Orientale, Novara, Italy; ^6^Department of Human Neurosciences, Sapienza University of Rome, Rome, Italy; ^7^Neuromed Institute IRCCS, Pozzilli, IS, Italy; ^8^Department of Clinical and Experimental Medicine, University of Messina, Messina, Italy; ^9^Neurology Unit, Department of Medical Area, ASST Pavia, Voghera, PV, Italy; ^10^Neurology Unit, San Filippo Neri Hospital ASL Roma 1, Rome, Italy; ^11^Parkinson's Disease and Movement Disorders Unit, IRCCS Mondino Foundation, Pavia, Italy; ^12^IRCCS Ospedale Policlinico San Martino, Genova, Italy; ^13^Department “G.F. Ingrassia”, Section of Neurosciences, University of Catania, Catania, Italy; ^14^Department of Advanced Medical and Surgical Sciences, Università della Campania “Luigi Vanvitelli”, Naples, Italy; ^15^Neurology Department, S.Eugenio Hospital, Rome, Italy; ^16^Department of Economics and Statistics, University of Salerno, Fisciano, SA, Italy

**Keywords:** essential tremor, family history, quality of life, genetic, aging

## Abstract

**Background:**

To date, there are no large studies delineating the clinical correlates of “pure” essential tremor (ET) according to its new definition.

**Methods:**

From the ITAlian tremor Network (TITAN) database, we extracted data from patients with a diagnosis of “pure” ET and excluded those with other tremor classifications, including ET-plus, focal, and task-specific tremor, which were formerly considered parts of the ET spectrum.

**Results:**

Out of 653 subjects recruited in the TITAN study by January 2022, the data of 208 (31.8%) “pure” ET patients (86M/122F) were analyzed. The distribution of age at onset was found to be bimodal. The proportion of familial cases by the age-at-onset class of 20 years showed significant differences, with sporadic cases representing the large majority of the class with an age at onset above 60 years. Patients with a positive family history of tremor had a younger onset and were more likely to have leg involvement than sporadic patients despite a similar disease duration. Early-onset and late-onset cases were different in terms of tremor distribution at onset and tremor severity, likely as a function of longer disease duration, yet without differences in terms of quality of life, which suggests a relatively benign progression. Treatment patterns and outcomes revealed that up to 40% of the sample was unsatisfied with the current pharmacological options.

**Discussion:**

The findings reported in the study provide new insights, especially with regard to a possible inversed sex distribution, and to the genetic backgrounds of “pure” ET, given that familial cases were evenly distributed across age-at-onset classes of 20 years. Deep clinical profiling of “pure” ET, for instance, according to age at onset, might increase the clinical value of this syndrome in identifying pathogenetic hypotheses and therapeutic strategies.

## Introduction

After the release of the new tremor classification by the International Parkinson and Movement Disorder Society (IPMDS) in 2018, essential tremor (ET) has been redefined as an isolated action tremor syndrome of the upper limbs with a duration of at least 3 years without other neurological signs (1). A novel construct of ET-plus has been proposed to classify those patients who, in addition to the core phenotype of ET, also manifest with rest tremor or with additional “soft signs” of uncertain relationship to the tremor syndrome (1). Moreover, new diagnostic categories of focal tremor of the head/voice and task-specific/position-dependent tremor, which were formerly included in the definition of ET, have been proposed (1). These notable departures from previous definitions of ET were aimed at improving the phenotypic homogeneity of these patients, given previous failures to identify robust pathophysiologic and etiologic correlates of ET with its loosely defined boundaries (2, 3). Although the distinction between ET and ET-plus might theoretically serve the purpose of creating homogenous groups of patients for subsequent studies (4), distinguishing between ET and ET-plus by clinical distinction alone and in the absence of clear objective biomarkers is very complex (5, 6). Nonetheless, the revision of the nosology of tremor syndromes by the 2018 IPMDS tremor classification, especially with regard to ET, conveys the implication that previously obtained information about the clinical features and natural history of ET needs to be tested (7). Several attempts have since been pursued to retrospectively re-classify patients according to the new classification and to provide a comparison between “pure” ET and ET-plus (7–11). They showed that ET-plus would be commoner, would have a higher age at onset (AAO), and would have a worse progression than ET (7–11), and these results mirror those obtained by a recent longitudinal study on 37 patients with “ET,” of whom more than 80% had, in fact, ET-plus at baseline (12). However, all these studies emphasized ET-plus and did not provide a detailed account of “pure” ET. In fact, no studies have attempted, to the best of our knowledge, to delve into the entity of “pure” ET diagnosed according to the new classification and to detail its clinical features. It might be possible that the removal of ET-plus as well as of focal and task-specific tremor from the group of “pure” ET, as suggested by the 2018 IPMDS consensus (1), would disprove some of the old concepts about ET in general, and this needs to be formally tested.

The ITAlian tremor network (TITAN) is a multi-center data collection platform (13), aiming to prospectively assess the phenomenology and natural history of tremor syndromes and to serve as a basis for future etiological, pathophysiological, and therapeutic research. For the current study, the aim of which is to provide a detailed account of “pure” ET, further in relation to such sensible anchors as AAO and the presence of family history (FH), we have extracted from the TITAN database the clinical data of patients with a diagnosis of “pure” ET according to the 2018 IPMDS criteria. We here present a cross-sectional analysis of the baseline data of patients with “pure” ET and describe its clinical features and its impact on the activities of daily life (ADL) and the quality of life (QoL).

## Methods

The protocol and preliminary findings of the TITAN study have been published elsewhere (13). In brief, patients aged > 18 years with any tremor syndromes, but the ones combined with Parkinsonism, were eligible for recruitment (13). Patients were prospectively recruited, and the diagnosis of ET, or otherwise, was based on the current tremor classification (1). Specifically, investigators were asked to indicate the tremor diagnosis according to the 2018 IPMDS consensus for every recruited patient and to list, in the case of ET-plus, which soft signs were present. For the current study, we extracted from the TITAN platform data of patients with a diagnosis of “pure” ET, therefore excluding patients with ET-plus, focal tremor, task-specific/position-dependent tremor, or any other tremor diagnosis.

All patients were assessed with a standardized protocol (13) including a structured interview to gather the following variables: sex, age at examination, self-reported AAO, presence of FH for tremor or for other neurological diseases, tremor distribution at onset, task-specificity at onset, and presence of sensory trick at evaluation. The presence of FH was operationalized as the existence of tremor (or other neurological disorders as detailed below) in either first- or second-degree family members (14), with the percentages reported in this study representing cumulative figures. As per the protocol, the history of any type of tremor would satisfy a positive FH, regardless of its distribution or putative diagnosis (e.g., ET or other tremor diagnoses), with the exception of tremor in the context of a diagnosis of Parkinsonism, which instead qualifies for FH for “other neurological disorders”. The latter information was collected to test the hypothesis of the possible clustering of different disorders.

Patients were assessed with the Essential Tremor Rating Assessment Scale (TETRAS) (15) which is composed of two parts: the performance subscale, with its total score providing an index of tremor severity, and the ADL subscale depicting tremor impact on ADL. Using individual items of the TETRAS performance subscale, we calculated the following variables: (1) “postural tremor” as the mean of the scores assigned to the items assessing upper limb tremor in the forward outstretched and lateral “wing beating” positions; (2) “kinetic tremor” as the mean of the scores assigned to the items assessing upper limb tremor during the finger-nose test and the Archimedes spirals drawing; (3) the “postural-kinetic ratio” computed as (“postural tremor” – “kinetic tremor”)/(“postural tremor” + “kinetic tremor”); and (4) the asymmetry index as (right-sided items – left-sided items)/(right-sided items + left-sided items). To depict the craniocervical involvement of tremor, we summed the proportion of patients with neck, face, or voice tremor. Finally, patients were asked to report on a scale from 0 (worst possible) to 100 (best possible) their perceived level of QoL.

After the release of the first protocol and the beginning of the research activities (13), an amendment was approved to gather information about the current and past pharmacological treatment. As such, the following information is available only for a subset of the recruited sample (88/208; 42.3%). Investigators were asked to record all current treatments for tremor with daily dosages and the corresponding patient-global impression of change (P-GCI) on an 11-point Likert-like scale with three explicit anchors (e.g., 0 = much better; 5 = no change; and 10 = much worse) (16). Similarly, investigators were asked to record previous treatments with the reason for withdrawal [e.g., inefficacy or presence of adverse events (AEs)].

The study has been approved by the ethics committee of the coordinator center (University of Salerno; study approval n.33_r.p.s.o._02/10/2020), and all subjects were requested to provide a written consent form to participate.

### Statistical analysis

We first examined each variable by computing some descriptive statistics (mean, quartile, variance, kurtosis, and skewness) in order to check their distribution and the presence of outliers.

For numeric variables, the *t*-test and ANOVA tests were performed for comparing the mean between two groups or more than two groups, respectively. Furthermore, in the case of more than two groups, we used Tukey's honestly significant differences to check for which groups the differences were significantly different. For categorical variables, a Pearson's chi-square test was performed. Unless otherwise stated, data are given as mean ± standard deviation (SD), the theoretical significant level α being fixed at 5%. All analyses were implemented using R (17), RStudio (18), ggplot2 (19), and psych (20) packages.

## Results

Out of 653 subjects recruited in the TITAN study by January 2022, the data of 208 (31.8%) patients (86M/122F) with “pure” ET were extracted for the current study. “Pure” ET represented the second most common tremor diagnosis after ET-plus (41.3%) and before tremor combined with dystonia (13.6%) (13).

**Figure 1:**
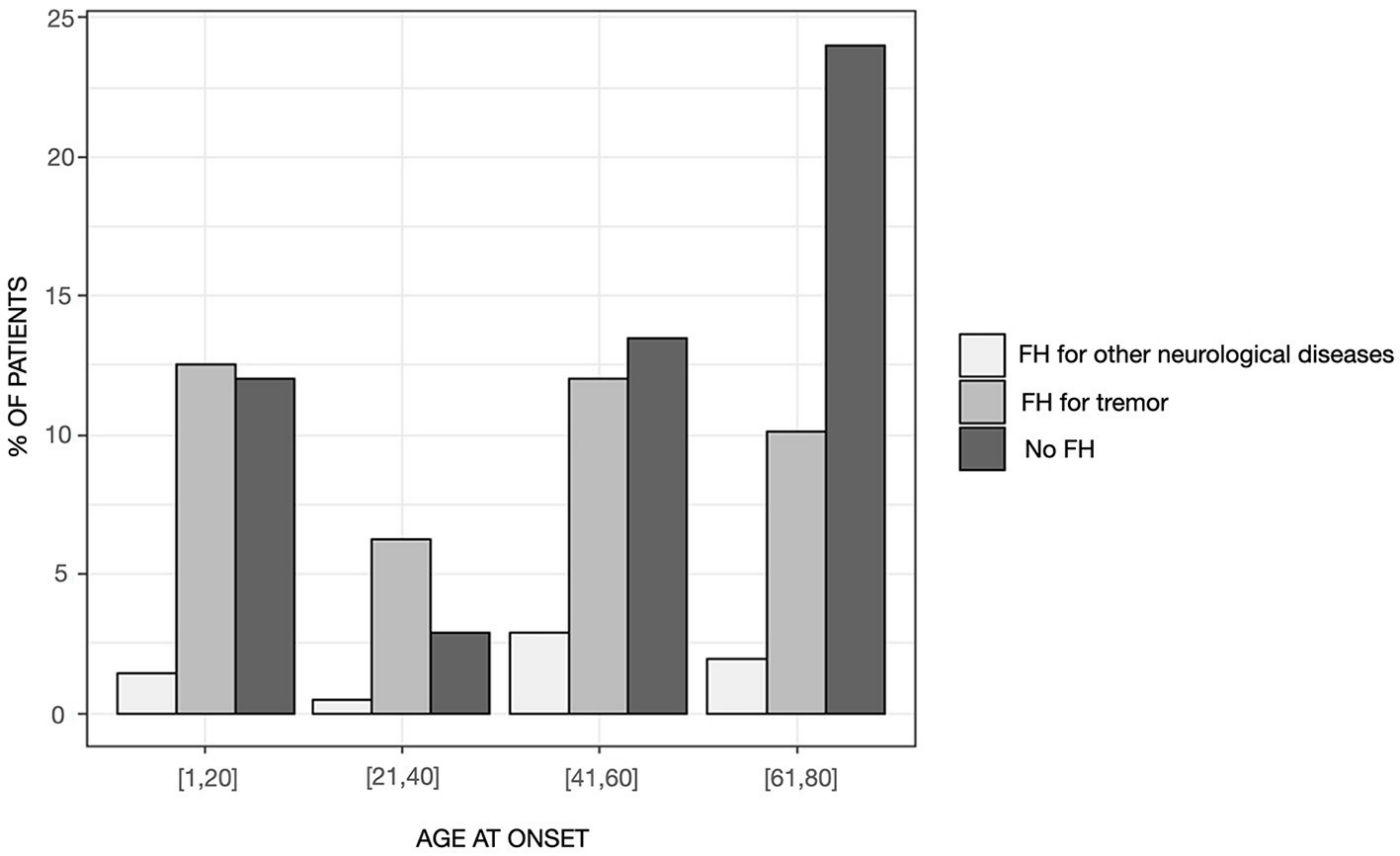
Distribution of patients by the presence of family history in the entire sample stratified in age-at-onset classes of 20 years.

**Figure 2:**
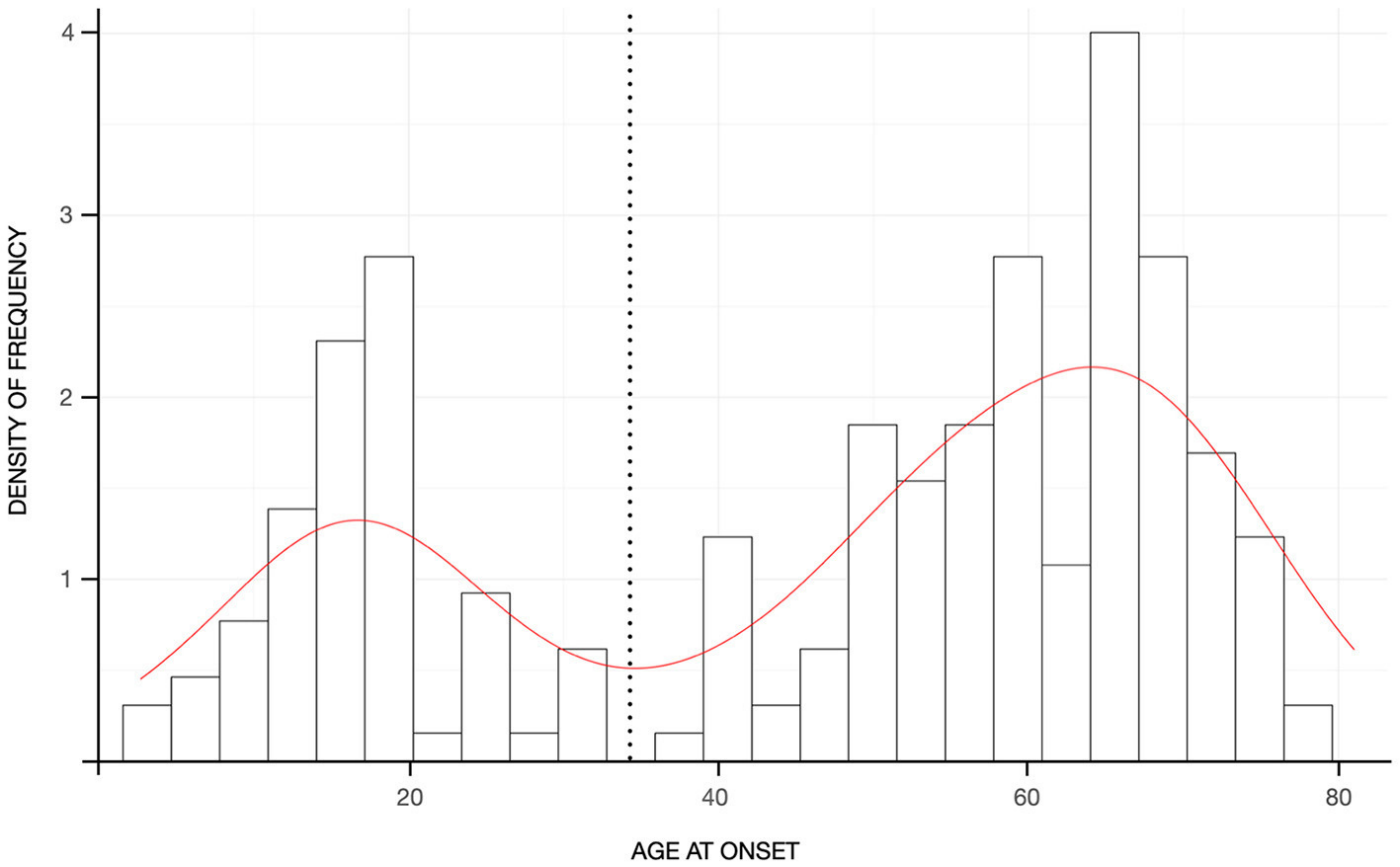
Histograms reflecting the density of frequency of patients according to the age-at-onset. The red curve represents the kernel density of the age at onset with the black dashed line reflecting the antimode.

Patients with “pure” ET had a mean age of 67.49 ± 12.27 years and a mean disease duration of 20.49 ± 20.27 years. Ninety-nine patients (47.6%) reported a positive FH for any neurological disorders, whereas the remaining patients were identified as sporadic cases. The distribution of familial cases by the AAO class of 20 years showed significant differences (χ2 = 13.563, *p* = 0.035), with sporadic cases representing the large majority of the class with AAO above 60 years and patients with a positive FH for tremor being evenly distributed across the classes although they were most represented in the class of age 21–40 years of AAO (Figure 1). The distribution of AAO was found to be bimodal, with the antimode being identified at 34 years (Figure 2). Accordingly, patients were stratified into two groups (e.g., early-onset <34 years and late-onset ≥ 34 years) for subsequent analyses.

**Figure 3:**
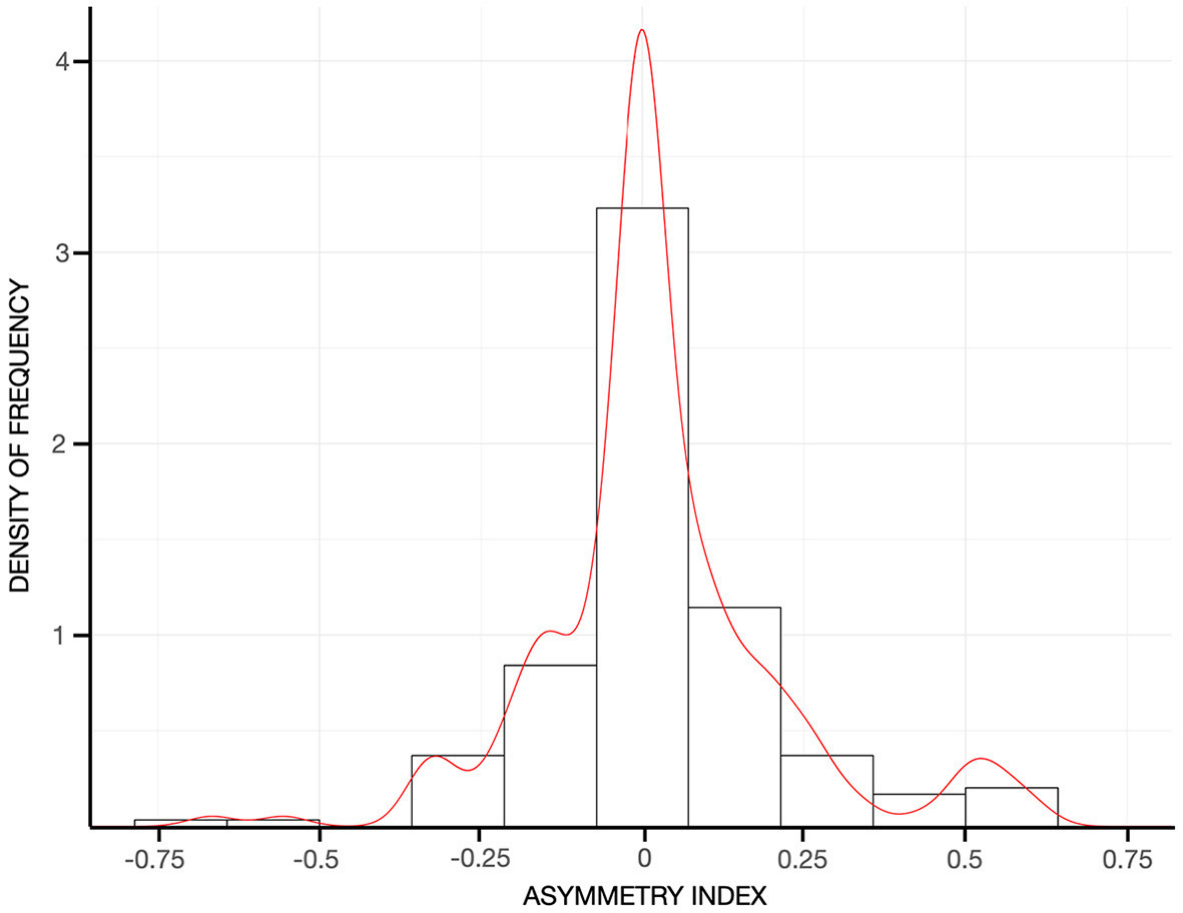
Histograms reflecting the density of frequency of the asymmetry index in the entire sample. The red curve represents the kernel density of the asymmetry index. Values close to 0, around which it peaks in the current cohort, indicating a symmetrical tremor between the two arms.

**Figure 4:**
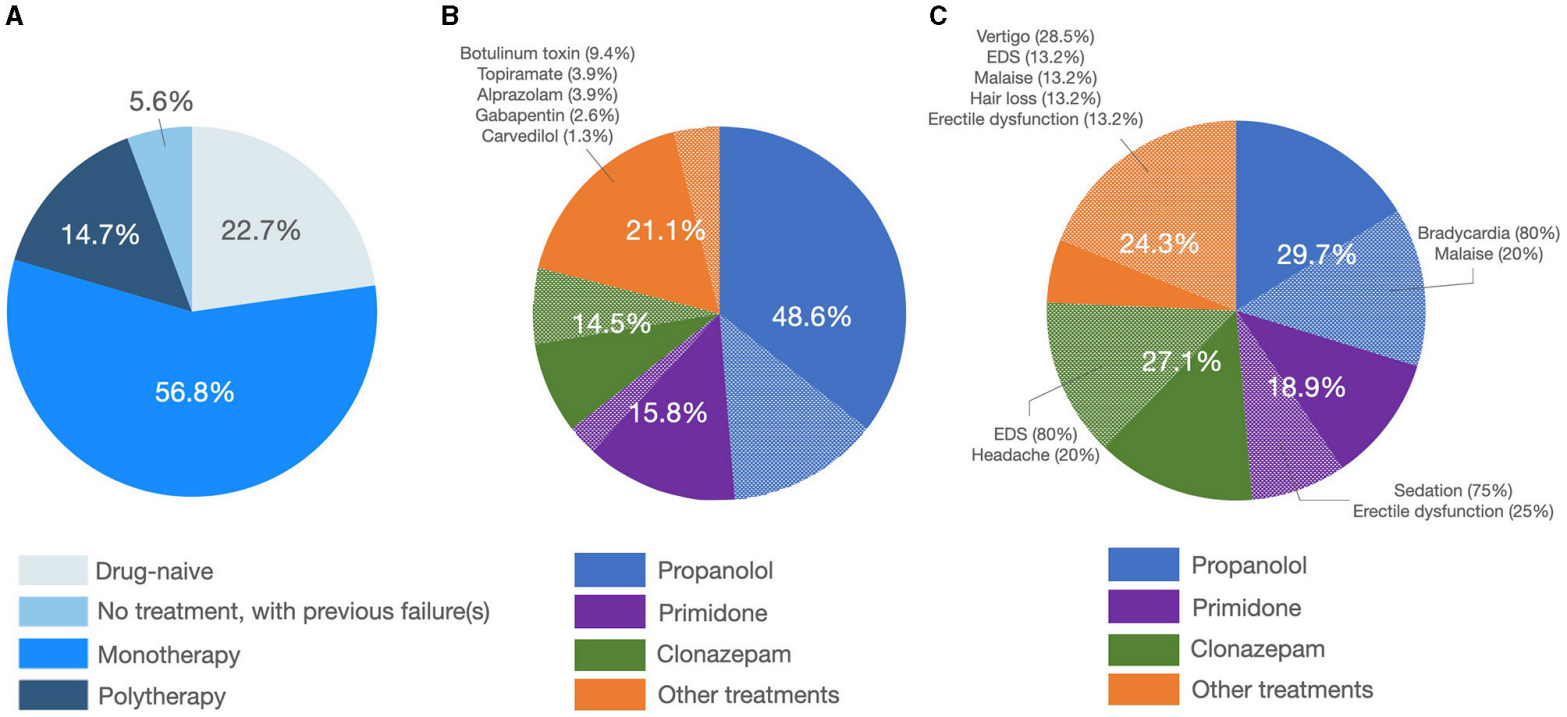
Treatment patterns and outcome. **(A)** The left pie graph shows the percentage of patients being on treatment or not. **(B)** The central pie graph shows the prescribed anti-tremor drugs with the plain slices representing patients reporting improvement/much improvement and dotted slices representing patients reporting no change/worsening in their tremor. **(C)** The right pie graph shows the percentage of patients discontinuing anti-tremor drugs because of inefficacy (plain slices) or the emergence of side effects (dotted slices, in which cases relative percentages are depicted).

In the entire sample, tremor was fairly symmetric (e.g., asymmetry index = 0.01 ± 0.35, Figure 3), and the postural component was more severe than the kinetic component (i.e., with a positive postural-kinetic ratio, Table 1). Tremor involved the cranio-cervical region in ~40% and the lower limbs in about 12% of cases. Task specificity at onset was reported by 16 patients (7.69%), and 16 additional cases (7.69%) were found to have a sensory trick at the examination. Table 1 details the demographic and clinical features of the entire sample. Treatment data were available for a subset of 88/208 (~42%). Over 70% of this subset was on one or more anti-tremor drugs, with only ~5% of these cases not being on any drugs because of previous failures (Figure 4A). The most commonly prescribed drug was propranolol (48.6%; mean daily dose of 64.17 ± 24.31 mg), followed by primidone (15.8%; mean daily dose of 155.21 ± 144.99 mg), clonazepam (14.5%; mean daily dose of 1.14 ± 1.15 mg), and other drugs (Figure 4B). Of patients on these treatments, 72.9, 83.3, 54.5, and 81.2%, respectively, were reported to be improved/much improved (Figure 4B). Approximately 42% of this sample further reported having previously discontinued other anti-tremor drugs either because of inefficacy (45.9%) or because of the emergence of AEs (54.1%). Figure 4C details the relative proportions of inefficacy or AEs for each anti-tremor drug.

**Table 1:** Demographic and clinical features of the entire cohort.

	**Entire cohort (*N* = 208)**
Age (years)	67.49 ± 12.27
Sex distribution (M/F)	58.65%/41.35%
Age at onset (years)	47.00 ± 22.72
Disease duration (years)	20.49 ± 20.27
FH for tremor (yes; %)	40.86
FH for other neurological diseases (yes; %)	6.73
Arm involvement at onset (%):	
- Absent	2.40
- Unilateral	22.60
- Bilateral symmetric	38.46
- Bilateral asymmetric	36.54
TETRAS, performance subscale	15.5 ± 6.85
Postural tremor	1.38 ± 0.60
Kinetic tremor	1.29 ± 0.69
Postural-kinetic ratio	0.05 ± 0.37
Asymmetry Index	0.01 ± 0.35
Cranio-cervical involvement (%)	41.35
Leg involvement (%)	12.98
TETRAS, ADL subscale	13.14 ± 8.18
QoL	70.53 ± 19.00

M, male; F, female; FH, Family history; TETRAS, The Essential Tremor Rating Assessment Scale; ADL, Activity of daily living; QoL, Quality of life. Data are reported as mean ± standard deviation or percentages.

### Comparisons between sporadic and familial cases

Out of 208 patients with ET, 85 patients (40.9%) reported a positive FH for tremor, 14 patients (6.7%) reported a positive FH for other neurological diseases including Parkinsonism and dementia, whereas 109 patients (52.4%) were sporadic cases.

AAO and age at evaluation were significantly different [ANOVA *F*_(2, 205)_ = 3.20; *p* = 0.043 and *F*_(2, 205)_ = 3.05; p=0.049, respectively], whereas disease duration was similar between the three groups. *Post-hoc* tests demonstrated that patients with FH for tremor had a significantly lower AAO (p=0.032; Table 2) and age at evaluation (*p* = 0.045; Table 2) than sporadic cases. Approximately 22% of cases with positive FH for tremor had leg involvement which was significantly higher than sporadic cases and cases with FH for other neurological diseases (Fisher's exact, two-tailed p=0.01, Table 2). No other differences were found between the groups in terms of clinical features at onset (e.g., onset site and presence of sensory trick and/or task-specificity), tremor phenomenology (e.g., postural-kinetic ratio and asymmetry index), craniocervical distribution, TETRAS performances, ADL subscales, and QoL (Table 2). No differences were observed in terms of treatment patterns or outcomes between the two groups (for all *p* > 0.05).

**Table 2:** Demographic and clinical features of the our cohort stratified according to the presence of family history for tremor or other neurological diseases.

	**NO FH (*N* = 109)**	**FH for tremor (*N* = 85)**	**FH for other neurological diseases (*N* = 14)**
Age (years)	69.20 ± 12.75	64.99 ± 11.75^a^	69.36 ± 9.15
Sex distribution (M/F; %)	61.47/38.53	58.82/41.18	35.71/64.29
Age at onset (years)	50.60 ± 23.45	42.36 ± 21.00^a^	47.14 ± 23.49
Disease duration (years)	18.61 ± 21.22	22.62 ± 18.24	22.21 ± 24.32
Arm involvement at onset (%):			
-Absent	4.59	0.00	0.00
-Unilateral	22.94	22.35	21.43
-Bilateral symmetric	34.86	41.18	50.00
-Bilateral asymmetric	37.61	36.47	28.57
TETRAS, performance subscale	14.91 ± 6.66	16.32 ± 6.90	15.11 ± 7.95
Postural tremor	1.33 ± 0.59	1.33 ± 0.60	1.10 ± 0.74
Kinetic tremor	1.28 ± 0.70	1.29 ± 0.70	1.38 ± 0.71
Postural-kinetic ratio	0.06 ± 0.32	0.06 ± 0.41	−0.08 ± 0.46
Asymmetry index	0.05 ± 0.35	−0.04 ± 0.35	0.07 ± 0.29
Cranio-cervical involvement (%)	36.70	44.71	57.14
Leg involvement (%)	8.26	21.18^a, b^	0.00
TETRAS, ADL subscale	12.45 ± 8.18	13.71 ± 8.31	15.07 ± 7.26
QoL	72.16 ± 19.21	69.65 ± 19.16	63.21 ± 15.01

M, male; F, female; FH, Family history; TETRAS, The Essential Tremor Rating Assessment Scale; ADL, Activity of daily living; QoL, Quality of life. Data are reported as mean ± standard deviation or percentages.

^a^Different from patients with no FH (*p* < 0.05).

^b^Different from patients with FH for other neurological diseases (*p* < 0.05).

### Comparisons between early-onset and late-onset cases

Sixty-six patients (31.7%) were classified as early-onset, whereas the remaining patients (142, 68.3%) as late-onset cases. Age at evaluation was significantly lower (61.00 ± 15.49 vs. 70.51 ± 9.02 years; *p* < 0.001), and disease duration was significantly longer (44.42 ± 18.11 vs. 9.37 ± 7.71 years; *p* < 0.001) in the early-onset than in the late-onset group (Table 3). The site of tremor onset was significantly different between the two groups (χ2 = 18.00; *p* < 0.001), with more than 90% of early-onset cases and ~65% of late-onset cases having a bilateral tremor of the upper limbs (Table 3). Tremor severity was higher in the early-onset than in the late-onset group (TETRAS performances subscale = 16.90 ± 6.12 vs. 14.85 ± 7.09; *p* = 0.034) as it was the postural tremor (1.49 ± 0.55 vs. 1.23 ± 0.61; *p* = 0.003), whereas kinetic tremor was not different (Table 3). The TETRAS-ADL score was not significantly different between the two groups, and the comparison only approached the statistical threshold (*p* = 0.058). The two groups were similar in terms of the remaining gathered variables (Table 3). No differences were observed in terms of treatment patterns or outcomes between the two groups (for all *p* > 0.05).

**Table 3:** Comparisons between early-onset (< 34 years) and late-onset (≥34 years) cases.

	**Early-onset (*N* = 66)**	**Late-onset (*N* = 142)**	***p*-value**
Age (years)	61.00 ± 15.49	70.51 ± 9.02	< 0.001
Sex distribution (M/F; %)	59.09/40.91	58.45/41.55	0.931
Disease duration (years)	44.42 ± 18.11	9.37 ± 7.71	< 0.001
FH for tremor	48.48	37.32	0.170
FH for other neurological diseases	6.06	7.04	1.000
Arm involvement at onset (%):			< 0.001
-Absent	0.00	3.52	
-Unilateral	7.58	29.58	
-Bilateral symmetric	54.55	30.99	
-Bilateral asymmetric	37.88	35.92	
TETRAS performance subscale	16.90 ± 6.12	14.85 ± 7.09	0.034
Postural tremor	1.49 ± 0.55	1.23 ± 0.61	0.003
Kinetic tremor	1.40 ± 0.72	1.24 ± 0.68	0.138
Postural-kinetic ratio	0.08 ± 0.40	0.03 ± 0.35	0.381
Asymmetry Index	−0.01 ± 0.23	0.02 ± 0.39	0.466
Cranio-cervcical involvement (%)	48.48	38.03	0.722
Leg involvement (%)	16.66	11.28	0.141
TETRAS, ADL subscale	14.74 ± 8.31	12.39 ± 8.04	0.058
QoL	69.09 ± 20.70	71.20 ± 18.19	0.576

M, male; F, female; FH, Family history; TETRAS, The Essential Tremor Rating Assessment Scale; ADL, Activity of daily living; QoL, Quality of life. Data are reported as mean ± standard deviation or percentages.

## Discussion

To the best of our knowledge, this is the first and largest multi-center study providing a detailed account of ET based on the 2018 IPMDS classification. On the one hand, our results broadly recapitulate previous findings, but it should be noted that nearly all available literature on the subject adopted former definitions of ET so comparisons should be taken cautiously. On the other hand, we also provide novel insights on “pure” ET, which indeed might stem from its new definition, as continued below.

Previous accounts generally found ET to be more prevalent in men, as confirmed in a recent meta-analysis (21), with only a few exceptions (11, 22). We observed a 1:1.5 ratio between men and women: this inverse proportion might be due to longer life survival in female elderly patients attending neurological consultation (11), but might also suggest sex-related differences in ET. The latter deserves to be tested in future worldwide epidemiological studies in view of the growing importance of understanding sex-related pathways to disease development (23).

As previously described (21, 24–26), frequency figures of ET steadily increased with advancing age, and we observed a bimodal distribution for the AAO with a peak in the second decade of life and a later increase in the sixth and seventh decades. As discussed in more detail below, this bimodal distribution seemed to reflect the effect of aging. Overall, a positive FH for tremor was reported in ~40% of the sample, which is lower than most previous reports of 60–75% (12, 25–27). This does not seem to be related to the difference in the criteria of operationalization for FH, as our criteria are arguably looser than those adopted in previous studies, which in most cases focused on the explicit diagnosis of ET in family members. One possibility is that the observed difference might be due to some sort of bias because of recruitment in single tertiary, university-based centers in most previous studies. In fact, our figure is in line with a study recruiting patients in three urban hospitals (25) as well as with the only available study that adopted the new IPMDS classification, yet retrospectively (11). Indeed, the possibility that the new definition of ET in itself has had an impact on these results cannot be ruled out. Interestingly, a small proportion of familial cases (~7%) were reported to have a FH for diseases other than tremor. This might suggest a possible clustering of different phenotypes including but not limited to ET (28). However, the possibility remains that these results are, at least in part, spurious given that parkinsonism and dementia are relatively common in the general population. Genetic testing of the TITAN cohort might shed light on this issue. Importantly, FH for tremor was evenly distributed across classes of AAO and did not account only for early-onset cases, whereas the impact of aging was preferentially observed for sporadic patients. This resulted in a lower AAO in familial than sporadic cases. Patients with and without family history were otherwise comparable for all other gathered variables, except for leg involvement in patients with FH for tremor. Given that disease duration was comparable between the groups, this result would reflect an intrinsic predisposition of patients with FH for tremor to develop leg tremor. This finding is in contrast to Chen et al. (27) who found leg tremor to be more common in patients with craniocervical tremor, longer disease duration, and higher tremor severity. It should be noted, however, that the latter results stem from research adopting former definitions of ET, thus likely including cases that we would currently label with ET-plus and that have been excluded from the current analysis which focused instead on “pure” ET. It has been shown that, applying the new tremor classification, leg tremor would be more common in ET-plus cases (8) who, in turn, also tend to have higher tremor severity (8, 11, 13). This might, therefore, explain the discrepancy between our and previous results. This consideration should be contemplated when examining all previously published literature as the new classification has likely had a great impact on the nature of the examined samples. Therefore, our results might set the “reference” according to the new definition of “pure” ET according to the 2018 IPMDS consensus and need to be further validated in other populations of different ethnicities.

Comparisons between early-onset and late-onset cases demonstrated some significant differences which, in view of the above, might be largely attributable to the longer disease duration in the former. Early-onset cases had, in fact, higher scores in the TETRAS performances subscale and in its postural items, which suggests that this is the tremor component prone to worsening over time. This, however, did not grossly impact ADL and QoL suggesting a relatively benign progression of “pure” ET in contrast to what has been shown for ET-plus (11, 12). Of note, some differences were identified between the two groups at onset, with the late-onset group reporting no or unilateral arm tremor at onset more frequently than the early-onset group. This might suggest that AAO is a useful anchor to identify clinically, and possibly biologically, different subtypes (25).

Overall, tremor phenomenology and distribution confirm most but not all previous studies (29). In our study, tremor was found to be largely symmetrical and more severe in its postural component. This is in line with what was generally assumed for ET (30) but is in contrast to Louis who suggested that the primary type of tremor in ET would be kinetic (29). Our figure of craniocervical tremor being present in ~40% of ET might appear slightly greater than previously reported in studies using the new definition of ET (11). However, we note that this might be due to the fact that we summed the proportion of patients with face, voice, and neck tremor. The reason for our choice stands in the fact that “midline tremor” has been suggested to be a proxy of greater clinical–pathological severity (10, 31). However, we did not find statistical differences in the frequency of midline tremor between patients stratified either according to the presence of FH or AAO although its frequency was higher in patients with early-onset and longer disease duration.

Importantly, we have reported the presence of some phenomenological features such as unilateral arm tremor and task-specificity at onset, and the presence of sensory trick at evaluation, which is generally assumed to be indicative of different tremor syndromes (32). These data might, therefore, turn useful in the attempt of calculating sensitivity/specificity values of these features against an alternative tremor syndrome (33), particularly of dystonia. These results further highlight one of the novelties of the new tremor classification, which is the possible diagnostic transition across tremor categories (1, 2). In fact, some of the patients reported here would have been diagnosed at onset as task-specific tremor to subsequently receive an ET diagnosis. Longitudinal analysis of this cohort will clarify whether these patients will develop overt dystonia.

Finally, we also report on treatment patterns and outcomes in our sample. On the one hand, they are in line with both evidence-based (34) and expert recommendations (35) about the treatment of ET, propranolol and primidone being the two most commonly prescribed drugs. On the other hand, we also observed a relatively frequent prescription of clonazepam, which was reported to be useful by at least 50% of patients on this drug. This contrasts with a small double-blinded trial that refuted the efficacy the clonazepam in ET (36). It should be noted, however, that we collected a patient-reported outcome and clonazepam might have been perceived as beneficial because of a primary effect on anxiety/embarrassment and of a secondary, indirect, effect on tremor. Approximately 10% of patients were receiving botulinum toxin injections for their tremor, ~5% of our sample were not receiving any drugs because of previous failures, and another one-third of patients reported no change or to be worse on the current drug regimen. This sums to ~40/45% of patients who are not satisfied with the current pharmacological options for ET and underscores the need for the development of new drugs for ET.

We acknowledge that our study has some limitations. First, we cannot entirely exclude a recruitment bias that is inherent to studies without a population-based design. However, the involvement of both secondary and tertiary movement disorder centers in the TITAN study (13) might have, at least in part, attenuated this risk, and therefore, our account of ET should be more realistic than those obtained in single-center studies. Second, we note that the diagnosis of tremor syndromes is made on a clinical basis given the lack of available biomarkers, and this might carry the risk of misdiagnosis in a proportion of patients. However, we strictly adhered to the current classification (1), and this represents the first study in which the diagnosis of ET according to the 2018 IPMDS tremor consensus has not been made retrospectively on the chart review. Moreover, the relatively long disease duration in our sample makes it unlikely that these patients have alternative conditions, including rare and/or treatable disorders (28), that usually present with combined tremor syndromes rather than with an isolated tremor syndrome such as ET. Third, we cannot exclude a certain degree of recall bias regarding the presence of FH. However, it has been demonstrated that the sensitivity of self-reported FH in patients with ET reaches ~85% (37). Similarly, self-reported AAO might also have suffered from a recall bias. However, prior studies have indicated that it is reliably reported by ET patients (38). We also acknowledge that alcohol responsiveness was not initially included in the study protocol since the 2018 IPMDS tremor consensus considered it to be unspecific for ET (1). However, this information has been included in a protocol amendment of the TITAN study, and we plan to compare different tremor syndromes for this feature in future reports. Finally, we acknowledge that information about past/current treatment and outcomes was available only for a subset of patients. However, missing data were equally distributed across the subgroups we analyzed (e.g., early- vs. late-onset and familial vs. sporadic cases), and therefore, this should not have biased the results in a major way.

In summary, we have here reported the clinical correlates of ET according to the new tremor classification. On the one hand, they broadly recapitulate the findings obtained by previous research using former definitions of ET, which might indirectly support the argument that the term ET-plus may be artificious. It is beyond the scope of the current study to add arguments to this debate, but we note that opposite experimental evidence favoring the distinction between ET and ET-plus is increasingly being produced (11, 13, 33, 39). On the other hand, the current study also provides novel insights with regard to a possible inversed sex distribution, the contribution of aging in late-onset cases, and the presence of some phenomenological features that might be characteristic of familial cases or have been classically attributed to different tremor syndromes. The observed differences with previous studies that adopted alternative operational criteria for ET further demonstrate the fulfillment of the primary intent of the 2018 IPMDS tremor classification to create a more homogeneous group of patients for future research. Our results would add that deep phenotyping of patients with “pure” ET might even better serve this purpose, and, in this regard, AAO seems to best identify distinct entities. Deep clinical phenotyping of ET, both of pure and plus forms, might increase the clinical value of this syndrome in identifying pathogenetic hypotheses, therapeutic strategies, and clinical research in the future.

## Data availability statement

The raw data supporting the conclusions of this article will be made available by the authors, upon reasonable request.

## Ethics statement

The studies involving human participants were reviewed and approved by Comitato Etico Napoli Sud. The patients/participants provided their written informed consent to participate in this study.

## Author contributions

RE, GL, AG, APi, LM, MB, CT, EO, CD, VM, FV, FD, AN, RD, LB, CS, AM, SB, GP, NM, EC, APa, AD, MR, and PB: conception and design of the study, or acquisition of data, or analysis and interpretation of data, drafting the article or revising it critically for important intellectual content, and final approval of the version to be submitted. All authors contributed to the article and approved the submitted version.

## Collaborators of the TITAN study group

Giulia Franco (Foundation IRCCS Ca' Granda Ospedale Maggiore Policlinico, Neurology Unit, Milan, Italy); Anna De Rosa (Department of Neurosciences and Reproductive and Odontostomatological Sciences, Federico II University, Naples, Italy); Lazzaro di Biase (Unit of Neurology, Neurophysiology, Neurobiology, Department of Medicine, Universit‘a Campus Bio-Medico di Roma, Rome, Italy); Marcello Esposito (Clinical Neurophysiology Unit, AORN Cardarelli, Napoli, Italy); Maria Chiara Malaguti (Neurology Department, S. Chiara Hospital, Trento, Italy); Raffaella Di Giacopo (Neurology Unit, Rovereto Hospital, APSS Trento, Italy); Roberto Ceravolo (Department of Clinical and Experimental Medicine, University of Pisa, Pisa, Italy); Francesca Spagnolo (Neurological Department, A. Perrino's Hospital, Brindisi, Italy); Marta Bianchi (UO Neurologia ASST Vallecamonica, Esine (Brescia), Italy); Roberta Vitaliani (Unit of Neurology, Department of Neuro-cardio-vascular, Ca' Foncello Hospital, Treviso, Italy); Laura Maria Raglione (Unit of Neurology of Florence, Central Tuscany Local Health Authority, Florence, Italy); Francesca Morgante, MD, PhD (Department of Clinical and Experimental Medicine, University of Messina, Messina, Italy; Neurosciences Research Centre, Molecular and Clinical Sciences Institute, St. George's, University of London, London, UK).

## References

[1] Bhatia KP Bain P Bajaj N Elble RJ Hallett M Louis ED. Consensus Statement on the classification of tremors. From the task force on tremor of the International Parkinson and Movement Disorder Society. Mov Disord. (2018) 33:75–87. 10.1002/mds.27121 29193359 PMC6530552

[2] Erro R Fasano A Barone P Bhatia KP. Milestones in tremor research: 10 years later. Mov Disord Clin Pract. (2022) 9:429–35. 10.1002/mdc3.13418 35582314 PMC9092753

[3] Espay AJ Lang AE Erro R Merola A Fasano A Berardelli A. Essential pitfalls in “essential” tremor. Mov Disord. (2017) 32:325–31. 10.1002/mds.26919 28116753 PMC5359065

[4] Erro R Picillo M Pellecchia MT Barone P. Diagnosis versus classification of essential tremor: a research perspective. J Mov Disord. (2023) 16:152–7. 10.14802/jmd.23020 37258278 PMC10236014

[5] Louis ED Bares M Benito-Leon J Fahn S Frucht SJ Jankovic J. Essential tremor-plus: a controversial new concept. Lancet Neurol. (2020) 19:266–70. 10.1016/S1474-4422(19)30398-9 31767343 PMC10686582

[6] Louis ED. “Essential tremor plus”: a problematic concept: implications for clinical and epidemiological studies of essential tremor. Neuroepidemiology. (2020) 54:180–4. 10.1159/000502862 32023613

[7] Prasad S Pal PK. Reclassifying essential tremor: implications for the future of past research. Mov Disord. (2019) 34:437. 10.1002/mds.27615 30653249

[8] Rajalingam R Breen DP Lang AE Fasano A. Essential tremor plus is more common than essential tremor: Insights from the reclassification of a cohort of patients with lower limb tremor. Parkinson Relat Disord. (2018) 56:109–10. 10.1016/j.parkreldis.2018.06.029 29958776

[9] Louis ED Huey ED Cosentino S. Features of “ET plus” correlate with age and tremor duration: “ET plus” may be a disease stage rather than a subtype of essential tremor. Parkinson Relat Disord. (2021) 1: 42–7. 10.1016/j.parkreldis.2021.08.017 34482193 PMC12766635

[10] Peng J Li N Li J Duan L Chen C Zeng Y. Reclassification of patients with tremor syndrome and comparisons of essential tremor and essential tremor-plus patients. J Neurol. (2022) 269:3653–62. 10.1007/s00415-022-10985-4 35094153

[11] Lolekha P Dharmasaroja P Uransilp N Sukphulloprat P Muengtaweepongsa S Kulkantrakorn K. The differences in clinical characteristics and natural history between essential tremor and essential tremor plus. Sci Rep. (2022) 12:7669. 10.1038/s41598-022-11775-8 35538158 PMC9091254

[12] Angelini L Paparella G De Biase A Maraone A Panfili M Berardelli I. Longitudinal study of clinical and neurophysiological features in essential tremor. Eur J Neurol. (2023) 30:631–40. 10.1111/ene.15650 36437695 PMC10107502

[13] Erro R Pilotto A Esposito M Olivola E Nicoletti A Lazzeri G. The Italian tremor Network (TITAN): rationale, design and preliminary findings. Neurol Sci. (2022) 43:5369–76. 10.1007/s10072-022-06104-w 35608737 PMC9385818

[14] Loens S Hamami F Lohmann K Odorfer T Ip CW Zittel S. Tremor is associated with familial clustering of dystonia. Parkinson. Relat Disord. (2023) 110:105400. 10.1016/j.parkreldis.2023.105400 37086575

[15] Elble R Comella C Fahn S Hallett M Jankovic J Juncos JL. Reliability of a new scale for essential tremor. Mov Disord. (2012) 27:1567–9. 10.1002/mds.25162 23032792 PMC4157921

[16] Hurst H Bolton J. Assessing the clinical significance of change scores recorded on subjective outcome measures. J Manipul Physiol Ther. (2004) 27:26–35. 10.1016/j.jmpt.2003.11.003 14739871

[17] R Core Team. R: A Language Environment for Statistical Computing. R Foundation for Statistical Computing, Vienna, Austria (2002). Available online at: https://www.R-project.org/

[18] RStudio Team. RStudio: Integrated Development for R. RStudio. Boston, MA: RStudio Team (2022). Available online at: http://www.rstudio.com/

[19] Wickham H. ggplot2: Elegant Graphics for Data Analysis. New York, NY: Springer-Verlag (2016). Available online at: https://ggplot2.tidyverse.org

[20] Revelle. psych: Procedures for Psychological, Psychometric, and Personality Research, R package version 2.3.6. Northwestern University, Evanston, IL, United States. (2023). Available online at: https://CRAN.R-project.org/package=psych

[21] Song P Zhang Y Zha M Yang Q Ye X Yi Q. The global prevalence of essential tremor, with emphasis on age and sex: a meta-analysis. J Glob Health. (2021) 11:04028. 10.7189/jogh.11.04028 33880180 PMC8035980

[22] Tallón-Barranco A Vázquez A Javier Jiménez-Jiménez F Ortí-Pareja M Gasalla T Cabrera-Valdivia F. Clinical features of essential tremor seen in neurology practice: a study of 357 patients. Parkinson Relat Disord. (1997) 3:187–90. 10.1016/S1353-8020(97)00031-X 18591074

[23] Mauvais-Jarvis F Bairey Merz N Barnes PJ Brinton RD Carrero JJ DeMeo DL. Sex and gender: modifiers of health, disease, and medicine. Lancet. (2020) 396:565–82. 10.1016/S0140-6736(20)31561-0 32828189 PMC7440877

[24] Louis ED Ferreira JJ. How common is the most common adult movement disorder? Update on the worldwide prevalence of essential tremor. Mov Disord. (2010) 25:534–41. 10.1002/mds.22838 20175185

[25] Hopfner F Ahlf A Lorenz D Klebe S Zeuner KE Kuhlenbäumer G. Early- and late-onset essential tremor patients represent clinically distinct subgroups. Mov Disord. (2016) 31:1560–6. 10.1002/mds.26708 27384030

[26] Lou JS Jankovic J. Essential tremor: clinical correlates in 350 patients. Neurology. (1991) 41 (2 (Pt 1)):234–8. 10.1212/WNL.41.2_Part_1.234 1992367

[27] Chen W Hopfner F Szymczak S Granert O Müller SH Kuhlenbäumer G. Topography of essential tremor. Parkinson Relat Disord. (2017) 40:58–63. 10.1016/j.parkreldis.2017.04.012 28442304

[28] Erro R Reich SG. Rare tremors and tremors occurring in other neurological disorders. J Neurol Sci. (2022) 435:120200. 10.1016/j.jns.2022.120200 35220114

[29] Louis ED. The primary type of tremor in essential tremor is kinetic rather than postural: cross-sectional observation of tremor phenomenology in 369 cases. Eur J Neurol. (2013) 20:725–7. 10.1111/j.1468-1331.2012.03855.x 22925197 PMC3511652

[30] Deuschl G Bain P Brin M. Consensus statement of the Movement Disorder Society on Tremor. Ad Hoc Scientific Committee. Mov Disord. (1998) 13 (Suppl. 3):2–23. 10.1002/mds.870131303 9827589

[31] Bologna M Berardelli I Paparella G Ferrazzano G Angelini L Giustini P. Tremor distribution and the variable clinical presentation of essential tremor. Cerebellum. (2019) 18:866–72. 10.1007/s12311-019-01070-0 31422549

[32] Erro R Rubio-Agusti I Saifee TA Cordivari C Ganos C Batla A. Rest and other types of tremor in adult-onset primary dystonia. J Neurol Neurosurg Psychiatry. (2014) 85:965–8. 10.1136/jnnp-2013-305876 24249781 PMC4145451

[33] Erro R Pilotto A Magistrelli L Olivola E Nicoletti A Di Fonzo A. A Bayesian approach to Essential Tremor plus: a preliminary analysis of the TITAN cohort. Parkinson Relat Disord. (2022) 103:73–6. 10.1016/j.parkreldis.2022.08.030 36063708

[34] Ferreira JJ Mestre TA Lyons KE Benito-León J Tan EK Abbruzzese G. MDS evidence-based review of treatments for essential tremor. Mov Disord. (2019) 34:950–8. 10.1002/mds.27700 31046186

[35] Ondo WG. Current and emerging treatments of essential tremor. Neurol Clin. (2020) 38:309–23. 10.1016/j.ncl.2020.01.002 32279712

[36] Thompson C Lang A Parkes JD Marsden CD. A double-blind trial of clonazepam in benign essential tremor. Clin Neuropharmacol. (1984) 7:83–8. 10.1097/00002826-198403000-00004 6367975

[37] Rocca WA Bower JH Ahlskog JE Elbaz A Grossardt BR McDonnell SK. Increased risk of essential tremor in first-degree relatives of patients with Parkinson's disease. Mov Disord. (2007) 22:1607–14. 10.1002/mds.21584 17546668

[38] Louis ED Schonberger RB Parides M Ford B Barnes LF. Test-retest reliability of patient information on age of onset in essential tremor. Mov Disord. (2000) 15:738–41. 10.1002/1531-8257(200007)15:4<738::AID-MDS1024>3.0.CO;2-4 10928590

[39] Brinker D Granert O Gövert F Tödt I Baumann A Zeuner KE. Grey matter correlates of dystonic soft signs in essential tremor. Parkinson Relat Disord. (2023) 112:105457. 10.1016/j.parkreldis.2023.105457 37245277

